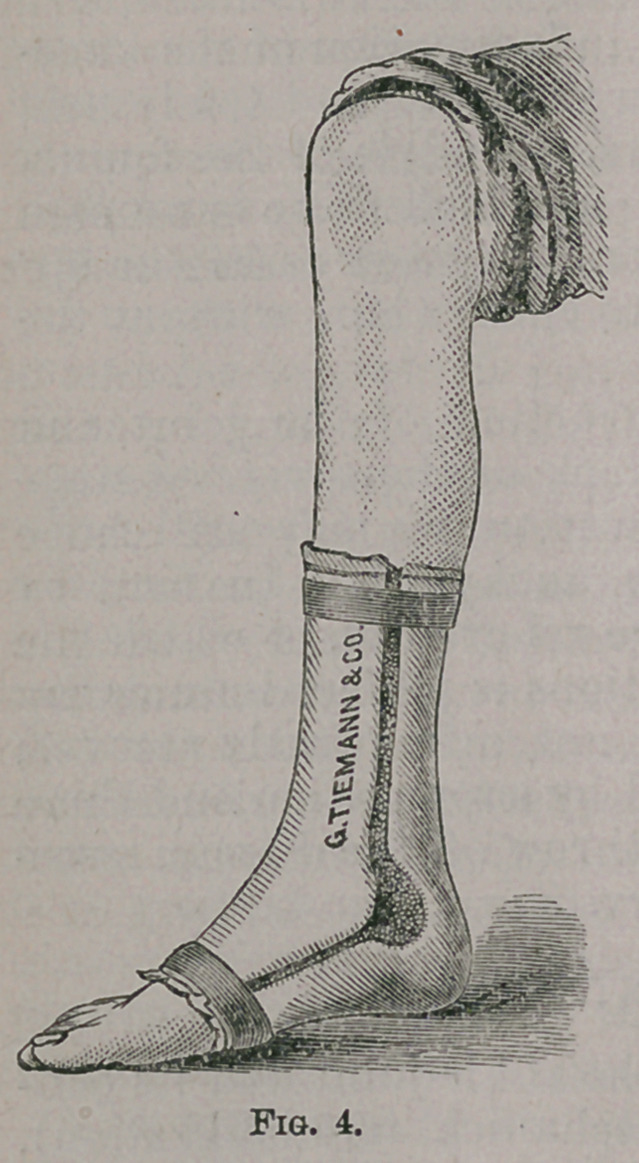# Mechanical Treatment of the Hip, Knee and Ankle-joints, by a Simple and Efficient Method

**Published:** 1879-06

**Authors:** Joseph C. Hutchinson


### Miscellaneous.

### Mechanical Treatment of Chronic Inflammation of the Hip, Knee, and Ankle-Joints, by a Simple and Efficient Method. The Physiological Method—with Cases.

BY JOSEPH C. HUTCHINSON, M. D.

I design in this paper to describe a plan for the mechanical treatment of inflammation of the hip, knee, and ankle-joints by methods which seem to me to be more simple, effective, and agreeable to the patient than those heretofore employed.

It may be stated at the outset that morbid conditions of the joints are, as a rule, essentially chronic, and whether the disease originates in the synovial membrane, the cartilages, bones, or investing fibrous capsule, ultimately the morbid action involves all the tissues, so that, without the previous history as a guide, it is often impossible to determine in what tissue the inflammation began. It is to my mind merely a pathological refinement, in many cases, of joint disease, especially in childhood, to attempt to describe the symptoms indicating distinct pathological states of the individual structures composing a joint. The treatment would be essentially the same whether one or all of the articular structures are simultaneously involved.

The indications for the mechanical treatment of inflammation of the joints of the lower extremities are to secure *immobility, extension, the removal of the superincumbent weight of the body, and means of enabling the patient to take open-air exercise.* The accomplishment of these indications, and the use of judicious medication and proper hygienic influences, comprehend all the principles of treatment.

*Immobility* of an inflamed joint, absolute and complete, is a primary and essential condition of its local treatment. The more effectually this is secured, the more rapidly and perfectly the joint recovers its normal condition, and the less danger there is of its being permanently damaged. The great obstacle to recovery is friction of the inflamed surfaces. I do not meean a mere limitation of the joint—such "rest" as is obtained by placing the limb upon a soft bed or pillow—but the perfect fixation secured by a splint or other means, which admits of no motion whatever. I am aware that many excellent surgeons believe that the danger of irreparable structural change and anchylosis of the joint is very great from prolonged fixation. This I am sure is an error. There may be a temporary anchylosis, such as arises from a diminution of the elasticity of the articular cartilages, and an enfeebling of the ligaments and muscles from disease; but such changes are, or need be, only temporary, for by careful and steadily increasing use, reparation takes place in all these structures, and after a time they show no defect. I have never seen true anchylosis when the joint has been immovably fixed until the inflammation has subsided, except in cases of extensive desfruction of the joint structures, in which case a cure by anchylosis is the thing to be desired. Exceptional cases no doubt occur, but the anchylosis takes place more commonly when fixation is complete, and more or less motion and friction are permitted before the inflammation has entirely subsided.

The object of extension is (1), to correct the mal-position of the limb. An inflamed joint is never straight; it involuntarily becomes flexed, nor is it possible for the patient to prevent or change this position. The flexion takes place slowly, almost imperceptibly, but surely, even when the limb has been permitted to rest quietly in bed undisturbed either by the patient or nurse; the degree of flexion depends upon the intensity or the duration of the disease. Every joint, when it becomes inflamed, assumes a characteristic *position* which it is important to know, not merely as a diagnostic sign, but also as a point which may be made useful in treatment. When the *hip-joint* is inflamed, the thigh is flexed on the pelvis, and, as a rule, is slightly adducted. The *knee-joint*, when inflamed, is always flexed more or less. In the case of the *ankle-joint*, the foot is flexed upon the leg, the heel is raised by the gastrocnemii, and the toes pointed downward. The *improper position* which the joint assumes should be corrected as soon as possible, even when the inflammation is acute; this is important in order that the different structures of the joint may not be kept in a state of undue pressure, or of inordinate tension, either of which interferes with healthy nutrition, and so conflicts with the curative process. As the joint becomes straightened under the influence of extension, the patient experiences an almost immediate diminution of pain. (2.) By means of extension we also overcome the spasm and contraction of the muscles, which, by reflex contraction, jam together the inflamed articular surfaces, and is the chief cause of pain in joint inflammations; but I do not believe it possible, by any amount of extension that can be applied, to separate the inflamed and swollen interior surfaces of the joint so as to relieve them from pressure and the consequent pain. What we do accomplish by extension is the relief of spasm and muscular shortening; and to quiet the muscles is an imperative therapeutic axiom.

The necessity for securing the beneficial effects of out-door air by means of some portative apparatus which removes pressure from the inflamed joint is now so generally appreciated that we need not urge its importance.

The special methods of meeting the above indications will be described when we consider the treatment of the diseases of particular joints.

### HIP-JOINT DISEASE.

The American Journal of the Medical Sciences for January, 1879, contains an article by the writer "On the Treatment of Morbus Coxarius by a New Method of Extension; the Physiological Method; with Cases," and I propose on this occasion to illustrate the method by exhibiting some patients who are now undergoing the treatment, and to show that the various kinds of portative apparatus now in use do not accomplish what is claimed for them. It is my purpose also to demonstrate that the "physiological method of extension" is quite as useful for the treatment of inflammation of the knee- and ankle-joints as it is for morbus coxarius.

For many years Harris, of Philadelphia, and others, treated morbus coxarius in bed by extension and fixation of the joint with the long splint formerly used for faacture of the thigh, with moderately satisfactory results; and in 1855, Dr. H. G. Davis, of New York, described a new portative apparatus designed to produce extension while allowing motion of the joint, and permitting the patient to enjoy the benefits of out-door exercise, so important in the treatment of this disease. It was claimed also by Davis and his followers that confinement to bed with the long splint applied, fixing the joint, not only impaired the general health, but increased the risk of anchylosis, which would leave the patient in a worse condition than if left to the tender care of Nature herself.

This new method of treatment awakened the interest of surgeons generally, and very soon afterward Sayre improved or modified Davis's instrument, and, with the enthusiasm of an ardent of nature, brought the new treatment prominently into notice, and by papers and lectures did more to secure its general adoption than the originator himself had done. The instruments of Taylor, Vedder, Washburn, and that devised by myself, are also modifications of Davis's, designed to accomplish the same indications, viz.: *mobility of the joint with extension.*

Barwell, Andrews of Chicago, Bauer, now of St. Louis, and Thomas, of Liverpool, believe that the indications for the proper treatment of the disease are to secure *immobility of the joint with extension,* and they have respectively devised very ingenious instruments to accomplish this purpose; while Professor Hamiltou's wire-gauze apparatus was designed merely to secure *immobility of the joint without extension.*

All these appliances are familiar to you, except that of Thomas, of Liverpool, which I will briefly describe. It consists of a flat piece of malleable iron, from three-quarters of an inch to an inch in width, by one-quarter in thickness, which extends from the lower angle of the shoulder of the affected side in a perpendicular line downward to the calf of the leg. A strap of hoop iron is riveted to the top of the upright, and nearly encircles the body a little below the axilla; another strap of iron, half the circumference of the thigh, is fastened to the upright just below the fold of the buttock; and a third, half the circumference of the calf, is riveted to the lower extremity of the upright. The instrument is carefully moulded to the inequalities of the body by means of wrenches, and is well padded and covered with leather. The apparatus having been applied, the patient is allowed to walk on crutches with a patten on the sound foot, so as to elevate the diseased limb two or three inches from the ground.

This apparatus will not allow tbe patient to sit down, and renders defecation very inconvenient.

We have therefore three classes of portable appliances in use for the treatment of morbus coxarius, all of which, with due respect to the ingenuity of their respective authors, I feel called upon to condemn: (1), because they do not achieve the objects for which they are designed; and (2), if they did, they are cumbrous and uncomfortable, and therefore should be abandoned, because the same indications can be accomplished by a method simpler and more comfortable to the patient.

The theory that motion and extension are obtained by the apparatus of Davis and his followers is a great deception. If you notice a patient wearing Taylor's or Sayre's long splint (modifications of Davis's), those most frequently used here, you will see that when he walks the whole pelvis swings, and there is no motion at the hip-joint.

This immobilization of the joint a kind Providence has secured, in spite of the efforts of the surgeon to prevent it. You will also observe that there is no extension made in the instrument, as the inventors claim, because the strap which is designed to produce extension, and passes from the ends of the adhesive plaster beneath the extension bar, is slackened at every step. This I have noticed lately in a number of cases in one of the hospitals of the city of New York, where there is a large orthopædic ward under the care of an accomplished orthopædic surgeon, who uses Taylor's apparatus. The instrument merely transfers the weight of the body from the hip-joint to the perineal band, but the extension is made by the weight of the limb alone.

The apparatus of Andrews, Barwell, and Bauer are equally inefficient in securing the objects for which they were designed; viz., to render the joint immovable and to produce extension of the limb. Thomas's instrument, by its long leverage, extending from the angle of the scapula to the calf of the leg, has some control over the movements of the joint; but it is unnecessary for this purpose, and, as already indicated, is very inconvenient to the patient; while the wire-gauze apparatus of Prof. Hamilton can have but little influence in producing immobility, because it does not extend far enough above and below the joint.

Why is it, then, it may be asked, if the appliances referred to are inefficient to accomplish what is claimed for them and are deceptive, that so much improvement has been reported from them when compared with others not having their features? For my own part, I am in the habit of explaining these favorable results by the fact that the use of the instruments devised by American ingenuity has liberated patients from in-door constraint, and enabled them to live and move and exercise in the open air, instead of being treated in bed as was formerly done; and also from the fact that the principal indications, immobility and extension, are achieved in spite of the apparatus used.

We have already considered the indications for the treatment of hip-joint disease, and also for the treatment of inflammation of the knee- and ankle-joints. They are: (1), to secure immobility of the joint; (2), to make extension; (3), to take off the superincumbent weight of the body; (4), to provide means to enable the patient to take open-air exercise; and I desire to demonstrate that they can be accomplished with comfort to the patient and convenience to the surgeon by the simplest expedients.

The method of treating hip-joint disease which I commend to your attention, after having used it exclusively for the last two years, is illustrated on the little patient before you (Figs. 1 and 2). To the shoe of the sound limb a steel plate, corresponding to the sole of the shoe, is attached by upright rods two and a half or three inches in length, so as to raise the foot from the ground; it is the shoe ordinarily used for shortened leg. The elevated shoe and a pair of crutches constitute the apparatus. As the patient stands on his crutches the diseased limb is suspended. The shoe is high enough to prevent the toes of the affected side from touching the ground, and the sole should be covered with leather to avoid noise when walking.

Here are brief notes of the cases taken from the records of the Orthopædic Infirmary. The first case is that of Henry S., and the record was made by Dr. A. R. Paine.

He is five years old, and was brought to the dispensary for treatment Feb. 4, 1879. His mother states that he began to have trouble in his left hip-joint eight months previously; that he had had pain in the hip and also in the knee from that time to the present, increasing at night; and that for some time he had not been able to walk. An examination revealed the existence of well-marked morbus coxarius, as indicated by the following symptoms: considerable fulness in the gluteal region, obliteration of the gluteal fold, the thigh slightly flexed upon the body; there is apparent anchylosis at the hip-joint, the pelvis moving with the femur; the effort to move the joint produces great pain; there is also great pain on pressing the trochanter inward and when the foot is jarred. The elevated shoe and crutches were ordered for him.

He was seen a second time Feb. 21st. He used the shoe and crutches about a week. The first day he tried them he was run against and knocked down, falling of course upon the lame hip; he suffered a great deal from the injury, cried all night, and the flexion of the limb was greatly increased, but on getting upon his crutches in the morning the pain subsided and the limb gradually resumed its former position. Since that time he has had much less pain and goes about easily and comfortably. March 11th, the mother says the boy is doing "splendidly;" he has no pain day or night, and the position of the limb is good. The case is before you, and speaks for itself. There is every reason to suppose that it will progress satisfactorily.

FIG. 1.

CASE II.—Morbus coxarius (third stage) progressive improvement by the use of the elevated shoe and crutches.

This little boy, three years old, was brought to the Orthopædic Infirmary, Feb. 14th, 1879, and the record of his case was made by Dr. H. W. Rand.

FIG. 2.—Shows the Shoe alone.

The parents think the present trouble commenced when the child was six months old. He was tolerably well nourished, and gives no history of injury. When he began to creep it was noticed that he favored the right leg. Two months later swelling appeared around the hip-joint, most prominent in the groin, where it was opened by the family physician, discharging a thin yellowish fluid.

Since the child began to walk he has always borne the most weight on the ball of the foot, rarely allowing the heel to touch the floor, owing to flexion of the thigh on the trunk. He complained of very little pain until December of last year, since which time pain has been almost constant and referred to the hip-joint.

When presented at the infirmary, the thigh was flexed on the abdomen, foot inverted, pelvis drawn up on affected side, nates flattened, and gluten-femoral crease lowered. Movement of the thigh excited spasmodic action in all the muscles around the joint, producing apparent anchylosis of the hip-joint, the pelvis moving with the femur. Pressure of the head of the femur against the acetabulum and pressure behind the trochanter caused pain.

Ordered elevated shoe (2½ inches) for left foot, and crutches.

Patient returned March 4th. Has learned to walk with the crutches and has had no pain for the past week. When last seen, March 14th, he was still entirely free from pain. Movement excited less spasmodic action in the muscles around the joint, and the flexion of the thigh on the abdomen had somewhat diminished.

A point of interest in this case is the early period at which children may be taught to use the elevated shoe and crutches.

The third case I bring forward as an illustration of complete recovery from morbus coxarius (third stage), treated by the elevated shoe and crutches. This case has been fully reported in the January No., 1879, of *Hays' Journal,* from notes by Dr. Paine, and I will not repeat it here. An examination shows that the position of the limb and foot is perfectly normal; there is no shortening; the joint moves freely in all directions without pain: the most careful scrutiny reveals no evidence of disease, and he looks and feels well. He was under treatment at the infirmary for eight months, when his recovery was pronounced complete.

By the simple appliances shown upon the patients whom I have presented to you this evening we fulfil all the indications for the mechanical treatment of hip-joint disease, and I desire to emphasize the statement that whatever artificial appliances for fixation and extension may be added, they simply tend to increase the discomfort of the patient.

*Immobility,* which it is just as important to obtain in the treatment of inflammation of this as of other joints, is secured by reflex contraction of the peri-articular muscles, aided by intra-capsular effusion, and the voluntary effort of the patient to keep the joint at rest on account of the pain which motion produces. Fixation of the joint is one of the earliest and most characteristic conditions in morbus coxarius; and it is so marked, that when we move the limb, the pelvis moves with it; there is apparent anchylosis. This rigidity continues until nature says immobility is no longer necessary, she secures it better than we can by any artificial appliances.

In the later stages of the disease motion is desirable, and gradually, as the inflammation subsides, the muscles become relaxed, motion returns, and anchylosis is prevented, except in extensive destruction of the joint surfaces, in which case a cure by anchylosis is the thing to be desired.

*Extension* is made by the weight of the suspended limb, which is equal in weight to one-fifth of the whole body, is greater than the weight ordinarily employed for extension, and is quite sufficient to subdue the spasm of the muscles which crowd the head of the bone into the inflamed acetabulum and is the chief cause of the pain which the patient experiences. We all know how promptly contraction of the muscles of the extremities, in cases of cholera or from other causes, is overcome by forcible extension. The pain in the part is relieved not by separating the inflamed articular surfaces as has been claimed, for we cannot separate them to an appreciable extent by any amount of extension that can be applied. The extension not only relieves pain, but it corrects the *malposition* of the limb, whatever it may be, and prevents the deformity which would otherwise occur from contraction of the muscles or partial dislocation of the head of the bone. By means of the elevated shoe and crutches *the weight of the body is removed from the diseased joint and the patient can enjoy all the benefits of open-air exercise*, conditions so evidently necessary as to require no special consideration.

It seems to me probable that the method of extension here described is both more efficient and more agreeable to the parts concerned, by reason of being more gradual, equable, less arbitrary and constraining, and, therefore, exciting a less degree of reflex resistance than most other methods. There is a certain degree of instinctive, unconscious recoil in the mind of every patient, young or old, against all the various devices of constraint or imprisonment which a splint or apparatus implies.

This plan of treatment should be adopted at once, whatever the stage of the disease, and continued until the cure is completed, except in the comparatively rare form of arthritic coxalgia, where acute inflammation of the synovial membrane and other soft structures of the joint is suddenly developed, attended with great constitutional disturbance and excruciating pain, increased by the slightest movement of the limb or the shaking of the bed. In such cases it would be inappropriate at first. Until after the acute symptoms have subsided they should be treated in bed with the long spint and the weight and pulley, together with other appropriate remedies.

There may be cases in which it will be necessary to make extension at night, by the weight and pulley, to relieve the usual nocturnal pain, while the elevated shoe and crutches are used during the day, but I have not thus far met with any, even among those who had used the night extension, with some portative apparatus during the day, up to the time they came under my treatment.

The patient soon learns that relief from pain is obtained by suspending the diseased limb, and then he is glad to walk or stand on the crutches three or four hours daily. This appears to be sufficient to relax the muscles to such a degree that spasmodic contraction, with the accompanying pain, does not take place at night.

For children who are too young, and older persons who are too feeble to use common crutches, Darrach's wheeled crutch, or the ordinary go-cart, are admirable aids to locomotion. Darrach's crutch is the best, as it is so constructed that the patient may be partially suspended in the crutch, if necessary, by a perineal band, which prevents fatigue, and it is also lighter and more elegant in construction. The elevated shoe should be used with either instrument. If a case comes under treatment at so advanced a stage that resection is necessary, the elevated shoe and crutches should be used after the active symptoms following the operation have subsided, instead of adopting the usual practice of confining the patient to bed and using the weight and pulley.

### THE KNEE-JOINT.

From the diseases of the hip-joint we will descend to those of the knee; but we must take the metaphor in an anatomical, not a surgical sense; for the frequency with which inflammation occurs in the knee-joint, owing to its complicated mechanical machinery and its exposed position both in relation to atmospheric changes and liability to injury from violence, invests the subject with an interest to the surgeon quite as great, if not greater, than that which pertains to the hip-joint.

Fig. 3.

For the morbid conditions of the knee-joint the indications for treatment are in all respects the same as for inflammation of the hip-joint, with the addition of *compression* over the joint.

The knee is not, like the hip, surrounded by powerful muscles, which by their rigidity immobilize the diseased hip-joint. It is necessary, therefore, in the case of the knee, to bring to our aid some mechanical restraint in order to effect complete rest. To secure *fixation* of the knee-joint, I use splints made of hatter's felt, such as you see on the patient before you (Fig. 3). It consists of seven layers of cotton-cloth saturated with shellac, and well rolled together while hot. It is manufactured of this thickness specially for me, by Mr. Holley, of South Fifth Avenue, New York, and may be obtained from Tiemann, and I suppose other surgical-instrument makers. That ordinarily sold consists of but five layers of cloth, which for most cases is not firm enough. To give effectual rest to the joint, the splint should be of sufficient length, and wide enough to nearly surround the limb; it should extend half way up the thigh, and to a corresponding point below the knee. A shorter splint, merely wide enough to cover the posterior part of the limb, does not secure the complete immobility which I have insisted upon in the treatment of diseases of the joints, where absolute rest is demanded. The splint having been cut of the proper length and width (the material is easily cut with a sharp knife), and the limb covered with a stocking, the felt made pliable, preferably by dry heat in an oven or before an open fire, or by immersion in very hot water, is applied to the limb and covered quickly, and firmly with a bandage from below upwards, so as to mould it to all the inequalities of the surface. While the splint is being applied an assistant should make extension from the foot, so as to straighten the limb as much as possible in cases where the joint is flexed; but no violent effort should be made to reduce the malposition; this can usually be accomplished by the gradual, painless (physiological) extension made by the weight of the limb, to which we shall presently refer. The joint surfaces are morbidly sensitive to pain, which would be greatly increased if they were suddenly and forcibly pressed together in the effort to reduce the deformity at once. If the surgeons's hands are very sensitive to heat, he may handle the splint better by wearing a pair of cotton gloves wet in tepid or moderately cold water. So soon as the splint regains its inflexibility, and this it does very quickly, it may be removed, trimmed up, and holes punched an inch or an inch and a half from the front edges for lacings. The object in punching the holes a little way back from the edges is to permit the splint to be made smaller by cutting off the edges, so that pressure may be kept up as the knee diminishes in size. The splint should nearly meet in front, and be laced tightly as the patient can bear with comfort; all the benefits of elastic pressure may be secured by surrounding the knee with a layer of wool-wadding, which never becomes matted, never losses its elasticity, and is an extremely comfortable method of making pressure, if the patient should complain of discomfort from the splint. If in any case it is considered desirable to leave the top of the knee uncovered, a semi-circular piece may be removed from either side of the splint, windows may be cut at any point where there are fistulous openings which require dressing. The splint may be made more comfortable in warm weather by perforating it here and there with a punch. If the leg is rotated on its longitudinal axis with a tendency to inversion or eversion of the foot, this should be prevented by extending the splint down to the foot.

If the leg is flexed when the splint is first applied, and cannot easily be forced into a straight position, the angle of the splint should be changed from time to time, as the leg becomes straighter under the influence of extension by its own weight. This may be done by softening the posterior part of the splint by the applica-of a sponge dipped in hot water; a bandage should then be firmly applied, while extension is made upon the leg by the hands of an assistant. So soon as the splint hardens, the bandage is removed and the lacings tightened. The splint, although firmly applied, does not interfere with the straightening of the joint by the entension made by the weight of the leg.

I prefer the felt splint to one made of plaster-of-Paris, leather, or liquid glass, because, while it is equally firm, it is also lighter, adapts itself just as well to the inequalities of surface about the knee, is more easily applied, its angle may be changed without removing it from the knee, and it may be unlaced and opened to examine the parts, or even removed, without disturbing the joint.

By means of the knee-splint we not only fix the joint and contribute to correct its malposition, but we also make *compression* upon the part, which is a valuable therapeutic auxiliary in the management of these cases, and its importance must not be overlooked. Compression causes absorption of non-purulent effusions into the joint, removes the boggy, infiltrated condition of the connective tissue which surrounds it, protects the part and gives support to the relaxed ligaments and synovial membrane.

*Extension* is best accomplished by the use of the elevated shoe and crutches which have already been described in considering the treatment of hip-joint disease. (Fig. 1.) The weight of the suspended leg, which may be estimated as one-twelfth to one-tenth of the weight of the body (eight to ten pounds in a body weighing one hundred pounds), is quite sufficient to tire out the muscles, which by reflex contraction compress the already suffering tissues within the joint, increasing the pain and leading to interstitial absorption—in short, the muscles are restored to their length. By means of extension we also correct the malposition of the limb, which is usually contracted to an angle of 120°; but extension has not the slightest influence in separating the diseased articular surfaces, nor do I consider this necessary. This method of extension is so gradual and equable, and therefore so agreeable to the parts concerned that the muscles are persuaded to relax, if such an expession is permissible in this connection, instead of being irritated and stimulated to contraction.

The apparatus of Prof. Sayre for producing extension of the diseased knee-joint, as well as the appliances of H. G. Davis and Sherman, of Chicago, for the same purpose, are creditable to the inventive genius of their respective authors; but those of you who have used either of them must be aware of the skill and experience necessary to apply them properly, the constant attention they require to keep them suitably adjusted, and the discomfort to the patient produced by the irritating effects of the adhesive plaster by which they are attached to the limb. Moreover, the effects to produce forcible extension by these various devices excites resistance, and the patient, young or old, instinctively recoils from the attempt to overcome muscular contraction by an exertion of strength applied by means of an apparatus.

*The weight of the body being removed from the diseased joint* by the use of the elevated shoe and crutches, the patient should be kept out of doors as much as practicable, and if old enough to understand the rationale of the treatmeut, the importance of using the crutches three of four hours daily should be explained to him, and, if necessary, their employment enforced. Patients should also understand the importance of keeping the joint at rest. They not infrequently complain of the restraint of the splint, and secretly remove it themselves (I speak especially of dispensary patients), not because they really suffer pain from the position or confinement of the limb, but because they are afraid of losing the use of the joint. I mention this, not to induce you to shut your ears or disregard the complaints of patients—on the contrary, I think they always deserve attention—but to warn you against deceit from this cause.

There are many mild cases of chronic inflammation of the knee-joint characterized by slight effusion into the joint and tenderness on pressure over the lower part of the inner condyle of the femur, or at the inside of the head of the tibia, in which there is no pain on pressing the articular surfaces together. In such cases the application of the knee-splint is sufficient to effect a cure without the use of the elevated shoe and crutches.

When the disease has resulted in destruction of the joint and caries, either from the violence of the attack or the advanced stage of the disease when it came under observation, we may still hope to save the limb and secure a cure by anchylosis. In fact, by rightly carrying out the indications above referred to, of which the first in importance in all joint inflammations is perfect immobility of the part, the most unpromising cases not infrequently recover; but if the patient is becoming exhausted by suppuration and there is not sufficient reparative power left to throw off the disease, resection or amputation may become necessary.

### THE ANKLE-JOINT.

In the treatment of inflammation of the ankle-joint and its consequences, *perfect rest* of the parts (mechanical immobilization), and the *removal of pressure* from the diseased articular surfaces, is quite as important, and I may add quite as satisfactory as in the diseases of the hip and knee, and the indications may be met in the same way. Instead of the felt, I prefer to use for fixing the ankle, two splints made of plaster-of-Paris, because they adapt themselves better to the inequalities of the surface about this joint, one to be applied in front and the other behind, extending from the middle of the leg to the ends of the metatarsal bones, and wide enough to leave an interval of half an inch between the edges on the inner and outer side. The splint should be made of two thicknesses of Canton flannel with coarse meshes, or three thicknesses of coarse towelling cut of the proper length and width. One layer of cloth is laid upon a table and covered with liquid plaster of the consistence of cream, and spread smoothly with a table-knife. The other layers are then immersed in the plaster and applied evenly and smoothly over the first; and when both splints have been prepared, one is applied in front and the other behind, with the under surface of the first layer, which is not covered with plaster, next to the skin, and covered with a roller bandage firmly applied from below upward. The surgeon should now grasp the foot, and holding it at a right angle to the leg, make extension until the plaster hardens, which requires about five minutes. The bandage should then be removed and the splints surrounded by three or four strips of adhesive plaster, and the bandage reapplied more loosely. Windows may be cut in the plaster so as to allow any openings that may exist in the parts to be uncovered. (Fig. 4.)

Fig. 4.

In all cases of diseased ankle-joint, the heel is raised more or less by the contraction of the gastrocnemii, and the toes pointed downward, if it is permitted to pursue its own course, and it is important to overcome the contraction of the muscles and place the joint at rest with the foot in its normal relation to the leg, (1) to secure its proper position, should anchylosis takes place; and (2) to relieve the pain produced by the unremitting muscular contraction day and night.

To remove pressure from an inflamed ankle-joint, and to provide means for letting the patient get the benefits of the open air, is not less important than in the case of a diseased hip or knee-joint. To accomplish these essential indications, a variety of instruments have been devised; but they are liable to the same objections which have been found to the appliances used for producing extension of the knee joint. After an experience somewhat extended in the treatment of these affections, I have no hesitation in recommending the elevated shoe and crutches as the best and simplest method of making extension and removing pressure; it is just as effectual for the ankle as for the knee and hip-joints. The weight required is not great, and the weight of the foot is sufficient to overcome the muscular contraction.

If the foot, from long neglect, cannot at once be brought to a right angle with the leg, the splints should be renewed every five or six day, increasing the extension a little at each application, until the foot is brought into proper relations with the leg.

The advantages which the mechanical treatment here described possesses over that commonly employed in the management of the diseases of the lower extremities are:

1. It saves the surgeon the trouble and annoyance of applying and carfully watching the instruments in ordinary use, to see that proper extension is kept up and undue pressure prevented; while the patient's comfort is greatly promoted by dispensing with adhesive plasters, which irritate the skin and require removal from time to time, and also with the perineal band in the hip disease, which is a constant source of discomfort.

2. The spasmodic contraction of the peri-articular muscles is overcome by the gentle, persuasive, and painless (physiological) extension made by the weight of the limb for several days; whilst forcible extension, either by the ordinary portative instruments, or by the weight and pulley, irritates the muscles and stimulates them to resistance and contraction, which must be overcome by main force.

3. I am quite confident, judging from the experience thus far obtained, that the plan of managing diseases of the joints herein described will shorten their duration more decidedly than can be done by the older methods of treatment.

4. The apparatus (if so simple a thing deserves the name of apparatus), is inexpressive, and can be made by any ordinary mechanic.

In conclusion, allow me to say that it was with a good deal of reluctance that I ventured to condemn as useless or hurtful the appliances hitherto in use in the treatment of diseases of the hip, knee and ankle-joints, and to commend to professional notice new and simpler methods. I should not have had the audacity to do so, had not my convictions, based upon practical experience, have seemed so plainly to warrant the positions I have endeavored herein to maintain. These convictions have been strengthened also by the favorable opinions expressed of the treatment of hip-joint disease, since the publication of my paper upon the subject, by surgeons in different parts of the country, for whose judgment I have long been accustomed to entertain the highest respect.—*Medical Record.*

———:o:———

### Philadelphia County Medical Society.

At the meeting of February 12th, Dr. Benjamin Lee read an article on an adaptable porous jacket employed in cases of Pott's disease and spinal lateral curvature. This jacket is made of porous felt, and its design is the same as that of Dr. L. A. Sayre's plaster-of-Paris jacket, except that the latter is immovable, while Dr. Lee's is adaptable. The latter peculiarity gives it various advantages, in the opinion of the lecturer.

In the discussion which followed, Dr. H. H. Smith expressed his opinion that those who wrote most on the subject ignored greatly the anatomical relations of the spinal column, and were too purely mechanical in their ideas. He feared that there was such a thing as living too long, when he noticed that plans of treatment formerly well known have been lost sight of, and others renamed as novelties. Fifty years ago, in the practice of Dr. J. K. Mitchell, of Philadelphia, the speaker had seen almost the same apparatus for suspension by the head and shoulders as had been exhibited this evening. This plan was then used by Dr. Mitchell, with the promise of very excellent results, but in several instances in which it had been tried, under the speaker's observation, the result was not what had been anticipated by either the surgeon or the patient. Indeed, very little had been gained by suspension that was not also derived from the old treatment of confining the patient to bed.

Independently of the fact that treatment by extension is not new, and independently of its former results, the speaker would inquire of the lecturer how suspension by the armpits can stretch the spinal column. You may push the scapulæ as far up as the ears, and yet not stretch the spine. The axillary horns of the crutch-like splint devised by Pott were not intended to extend the spinal column, except by the instrument makers; but the axillary or crutch-head processes kept the spine from bending forward, and thus straightened it, while apparently increasing in length. Dr. Smith would also extend this objection to the practice of self-suspension, which, though it might stretch the pectorel and scapular muscles, could not possibly extend the vertebral column. The difference in height shown after suspension by the head is in some cases but slight, or may be an error in the measurement, but in other cases it is due to throwing the shoulders backward and partially unbending the low formed in the spinal curvature.

Dr. Lee replied at some length to the observations of Dr. Smith, stating that in the suspensory apparatus the shoulder straps are not intended to extend the spine, but merely to relieve the patient occasionally, by taking part of the weight from the neck in applying the jacket. When using self-suspension as an exercise, the axillary bands are never used.

Dr. M. O'Hara reported the case of a child, sixteen months old, who had for two days presented the following symptoms: intense pain, as if from violent colic; threatening convulsions and sleepiness. The bowels and bladder were thoroughly evacuated without effect. Relief was obtained only after narcotism by potassium bromide and opium. By accident the mother perceived a foreign body protruding from the anus, and by traction withdrew about eight inches of common grocery twine, which at once afforded complete relief.—*Medical and Surgical Reporter.*

## Figures and Tables

**Fig. 1. f1:**
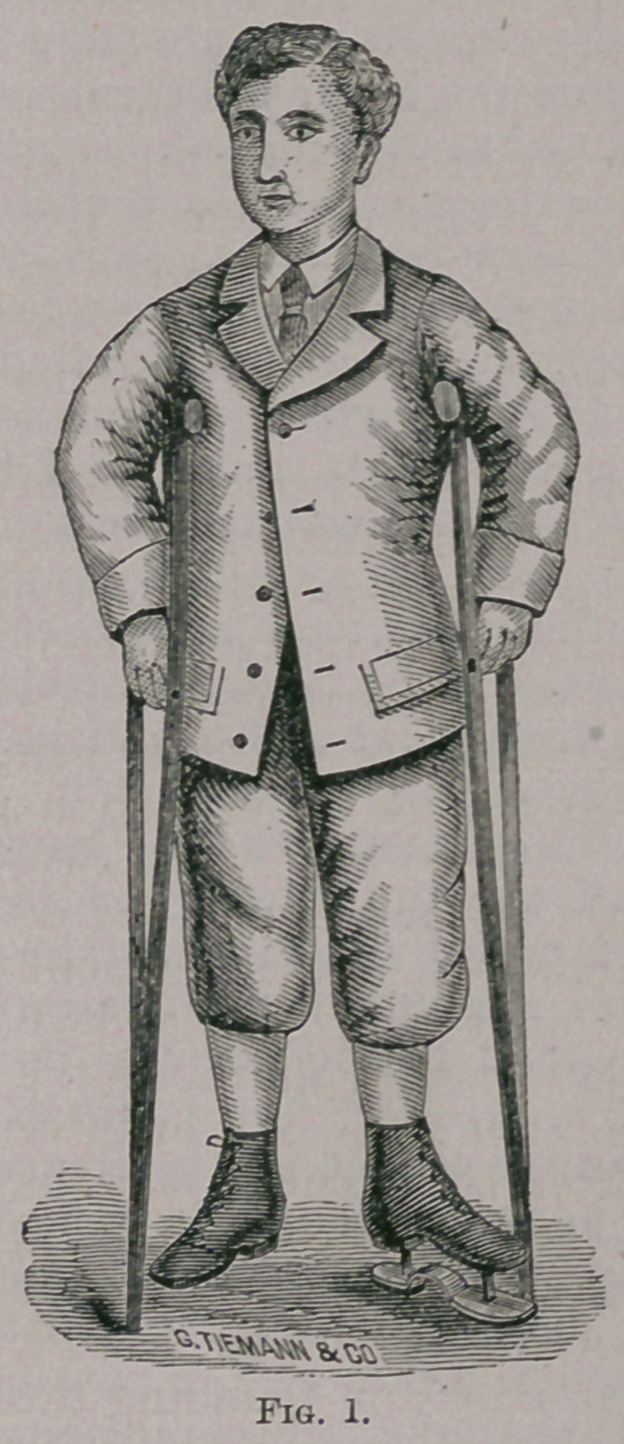


**Fig. 2. f2:**
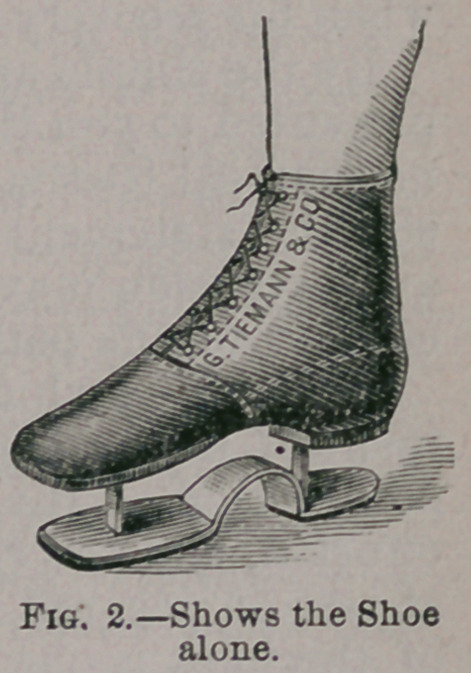


**Fig. 3. f3:**
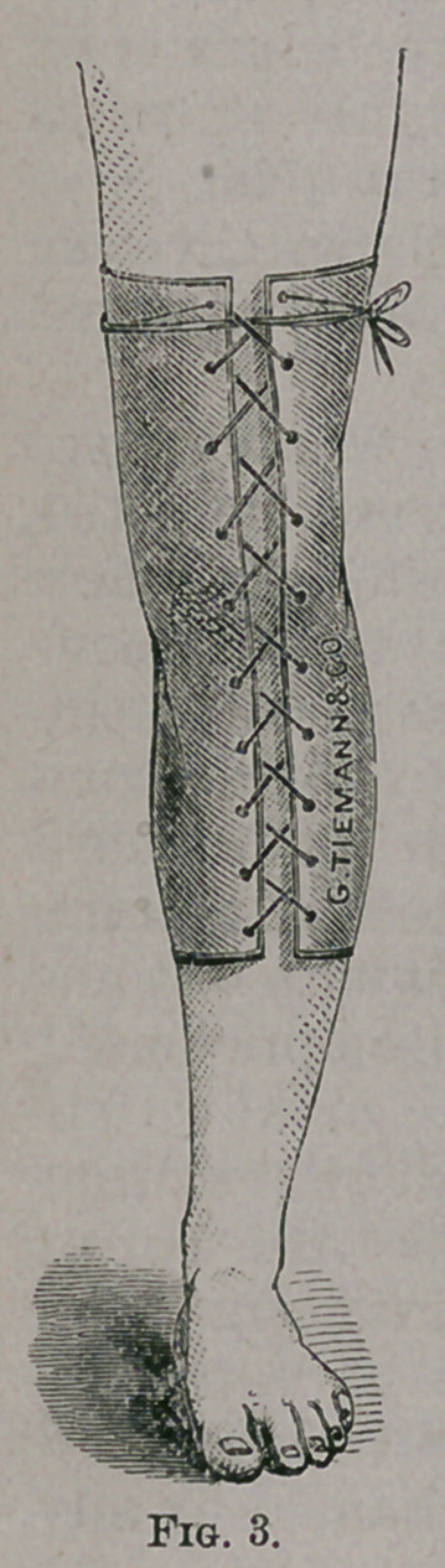


**Fig. 4. f4:**